# What Are the Duties of a Publishing Committee?

**Published:** 1887-04

**Authors:** 


### WHAT ARE THE DUTIES OF A PUBLISHING COMMITTEE?

Our distinguished contemporary of the *Journal of the American Medical Association* is hypercritical in the notice of the Texas

Transactions, which notice we copied in December, in connection with others. Moreover, considering that there are five pages of index to the Transactions, (to which our esteemed contemporary of the *Southern Practitioner* alludes as "full and comprehensive,") it is unjust to deprecate, as the JOURNAL does, the entire absence of an index. No doubt an *analytical* index would have been better; but, the covers of the Transactions having been ordered for a six hundred page book, and the amount of manuscript exceeding the printer's estimate, it was difficult to make them hold the one hundred pages more; and it would have been simply a physical impossibility to get in a more extended index.

As there is no rule for "editing" the Transactions, the publishing committee must be governed by precedent, or follow their own judgment. The publishing committee in this instance exercised their judgment, and we are sorry not to have the approval of our wise contemporary. We evidently differ as to the latitude to be exercised by the compiler of Transactions. We hold that it does not extend to altering or changing the titles of papers, which have been selected by their authors; nor to changing the construction or phraseology of any paper. To do so would entail an amount of time and labor which no members would be willing to assume; it would be tantamount to re.writing them; and as to proper names, all editors know the difficulty here presented; manuscript must be well and carefully prepared, and all proper names must be plainly written, (which is seldom the case) before mistakes in this particular can be justly chargeable to the publishing committee.

Indeed, it may be fairly questioned, whether a publishing committee has a right to correct *any* errors—of whatever kind, except purely clerical errors—in MMS. furnished by members for publication; for there are differences in degree of scholarship, as well as in scientific attainments amongst the members; and if the publishing committee were to correct all defects in papers thus presented, all the members would be put upon an equal footing; and the Transactions would be a fraud; it would not be a reflex of the Association, but of the Publishing Committee.

*Gaillard's Journal* also finds fault of our having put all the papers in an appendix. We surely see no reason why they should not be put there, in as much as the volume purports to be a record of the "transactions," (which are given in detail) and followed by an appendix of all papers and documents. It must be remembered in this connection, that many of the papers were not read, either in the sessions, or sections; some, not even by title; and such do not belong properly in the proceedings, but in an appendix—they pertain to the proceedings, yet are not strictly a part of the proceedings. Nor can we understand what *Gaillard's Journal* means by saying the "absence of an index is a serious fault," in as much as the volume has an index embracing the subject matter of the entire volume—an index which others have commended.

We make no apologies, and none are needed. Our members understand and appreciate the peculiar difficulties under which the publishing committee labored in bringing out this volume, and it is not necessary to explain it to our contemporaries; though it is to be hoped if they were in possession of the facts, they would, at least, be just.

While the above mentioned contemporaries have been hypercritical in the matter, we point with pride to, and gratefully acknowledge the kind and very complimentary remarks from their colleagues of the *New York Medical Journal*, the *Southern Practitioner*, the *Medical and Surgical Reporter*, the *American Lancet*, "*Progress*," and others. The external appearance of the volume, and the arrangement of the papers, "all in an appendix," is after that of the Georgia Transactions, which, edited and published by the scholarly Gray, is a model of neatness and good taste. Take the Transactions of Texas, all in all, and we a e not ashamed of a comparison.

We believe, with the exception of the two above named contemporaries, every journal in America has not only complimented the work, but has yielded the palm for excellence in mechanical execution; and, considering it was not published by a large publishing establishment, nor in New York, nor Philadelphia, but right here in Austin, by a job printing establishment, it is a great triumph, and more particularly when we consider that it cost only $1.55 per volume.

